# Species Diversity and Epidemiological Trends of Candida Isolates Recovered From Vaginitis Cases

**DOI:** 10.7759/cureus.114632

**Published:** 2026-08-16

**Authors:** Suman A Pawar, Geeta S Karande, Satish R Patil

**Affiliations:** 1 Department of Microbiology, Krishna Institute of Medical Sciences, Krishna Vishwa Vidyapeeth (Deemed to be University), Karad, IND

**Keywords:** candida albicans, chromagar candida, non-albicans candida, species identification, vulvovaginal candidiasis

## Abstract

Background

Vulvovaginal candidiasis (VVC) is the most prevalent cause of vaginitis among women of reproductive age. Although *Candida albicans* remains the predominant pathogen, an increasing prevalence of non-albicans *Candida *(NAC) species has been reported, necessitating accurate species identification for appropriate management. The aim of the present study was to determine the epidemiological profile and species diversity of *Candida* isolates from women with vaginitis and to characterize their identification using conventional phenotypic methods and CHROMagar *Candida*.

Methods

A descriptive cross-sectional study was conducted by the Department of Microbiology in collaboration with the Department of Obstetrics and Gynecology at Krishna Institute of Medical Sciences, Krishna Vishwa Vidyapeeth and Krishna Hospital and Medical Research Centre, Karad, India. A total of 428 high vaginal swab specimens were collected from women with clinically suspected vaginitis between reproductive ages. Samples were processed by direct microscopy, culture on Sabouraud’s dextrose agar, Gram staining, germ tube testing, carbohydrate fermentation testing, and CHROMagar *Candida* for species identification. Descriptive statistics were used to analyze the data and were presented as frequencies and percentages.

Results

Among 428 women with suspected vaginitis, 127 (29.67%) were culture-positive for *Candida* species. The majority of cases occurred in the 18-28-year age group (55.12%). Pregnant women accounted for 86 (67.72%) cases, while 41 (32.28%) were not pregnant. Species identification revealed *Candida albicans* as the most common isolate (47.24%), followed by *Candida krusei* (25.20%), *Candida tropicalis* (16.54%), and *Candida glabrata* (11.02%). Overall, NAC species made up 52.76% of the isolates. CHROMagar *Candida *enabled quick presumptive identification of *Candida *species based on their characteristic colony pigmentation, and the results corresponded with the criteria obtained through conventional phenotypic methods.

Conclusions

VVC remains a significant cause of vaginitis, particularly among young reproductive-age and pregnant women. Although *C. albicans* was the most common individual species, NAC species collectively accounted for a higher proportion of isolates, highlighting the substantial burden of NAC species and the importance of routine species-level identification. CHROMagar *Candida* may serve as a useful complementary presumptive phenotypic method for species differentiation in routine laboratory practice.

## Introduction

Vaginal *Candida *infections, also known as vulvovaginal candidiasis (VVC), are inflammatory conditions of the vagina triggered by *Candida *that typically affect the vulva concurrently with vaginitis [1]. Bacterial vaginosis (BV) is responsible for almost half of all instances of vaginal infection worldwide, in contrast to vaginal candidiasis, which is the subsequent predominant reason for this, impacting about 20% of those who are pregnant and 30% of non-pregnant women. Approximately 75% of women have at least one VVC episode while they are fertile [2].

Predisposing aspects such as socioeconomic status, hygiene standards, sexual behavior, diabetes mellitus, oral contraceptives, suppression of the immune system, and hormonal shifts during pregnancy are frequently linked to the incidence of VVC. Antibiotic consumption has been attributed to an increased occurrence of VVC. The vaginal lactobacilli, known as beneficial bacteria that shield the epithelial layer of the vagina, are destroyed by antimicrobial drugs. As a result, the potential of *Candida *populations adhering to the vaginal mucosa and colonizing the vaginal region could ultimately result in infection [3].

*Candida *infections are a highly widespread category of fungal infection among members of human populations. Although over 200 species of *Candida *have been identified, five of these species *C. albicans*, *C. tropicalis*, *C. parapsilosis*, *C. glabrata*, and *C. krusei *are responsible for around 90% of fungal diseases considered extensive in humans. Both morbidity and mortality caused by non-albicans *Candida *(NAC) strains are rising, despite *Candida* *albicans *continuing to be the species with the highest prevalence [4].

Whereas *Candida *species are members of the diverse endogenous vaginal microbiota, they proliferate quickly in conditions that induce typical morphogenesis, leading to prolonged urinary problems, pruritus, and copious amounts of "cottage cheese-like" secretion [5]. Complications brought on by *Candida *species are dependent on the development of virulence factors like adherence, phenotypic transition, development of biofilm, forming a germ tube, and the synthesis of hydrolytic enzymes [6]. *C. albicans* is probably the most common pathogenic agent, especially for the more serious chronic disease named recurrent VVC, even though several species of fungi in the genus *Candida *may result in acute VVC [7].

Nevertheless, the majority of VVC instances have no discernible manifestations. Pregnancy, compromised immunity, hormonal disturbances, and chronic diabetes are a few typical instances of causes of VVC [8]. Expectant mothers are more prone to VVC infections because of a spectrum of metabolic factors that favor *Candida *proliferation. Elevated estrogen concentrations, elevated vaginal glycogen formation, and immunologic disturbances are some of these alterations that encourage the growth and continued existence of species of *Candida *in the mucous membrane of the vagina [9].

*Candida *species found in clinical specimens can be differentiated more quickly using enzyme-based reaction techniques with chromogenic substrates. Species of *Candida *can be distinguished with excellent sensitivity and specificity using the chromogenic agar (CHROMagar *Candida*) medium. The majority of research studies have taken advantage of this practical and trustworthy approach [10].

BV, VVC, and trichomoniasis are the majority of common causes of vaginal manifestations. Despite the prevalence of vaginitis and vaginosis, many clinical settings struggle to make a reliable assessment [11]. In addition to causing morbidity in women, VVC has a detrimental effect on social and professional life and is increasingly recognized as a significant global health issue linked to direct or indirect monetary damages [12].

## Materials and methods

Study design and sample size

The present cross-sectional descriptive study was conducted in the Department of Microbiology in association with the Department of Obstetrics and Gynecology at Krishna Institute of Medical Sciences, Krishna Vishwa Vidyapeeth and Krishna Hospital and Medical Research Centre, Karad, from May 2024 to August 2025. The primary objective of the study was to identify and characterize the species distribution of *Candida *isolates recovered from women presenting with vaginitis.

During protocol development, sample-size requirements were estimated prospectively using the standard formula: n=4pq/L^2 ^where, p represents the anticipated proportion, q = 100 − p, and L^2^ represents the allowable error (precision), which was set at 10% (10^2^). The factor 4 corresponds approximately to a 95% confidence level. The sample-size assumptions were based on previously published literature by Babin et al. [13], as specified in the IEC-approved study protocol. Anticipated proportions of 90% for *C. albicans *and 78% for NAC* *were adopted for sample size calculations. These anticipated proportions were used for sample size planning and were not intended to represent the final observed species distribution. An anticipated proportion of 90% was used for *C. albicans* because using a proportion of 100% would result in q = 0 and would therefore not provide a usable sample size estimate using the 4pq/L^2^ formula.

Formula: n=4pq/L^2^

*Candida albicans* = 4×90×10/10^2^ = 36

Non-albicans *Candida *= 4×78×22/10^2^ = 69

During protocol development, these two-component sample-size requirements were combined to establish a planned overall target of 105 *Candida*-positive isolates for the single study, with the intention of ensuring adequate representation of both prespecified *Candida *categories. Consecutive sampling was employed throughout the study period. A total of 428 high vaginal swab (HVS) specimens were collected, of which 127 yielded *Candida *isolates, all of which were included in the final analysis.

Inclusion Criteria

Non-repetitive, clinically suspected patients with vaginitis within the reproductive age range of 18 to 45 years old, and married, pregnant, and sexually active women with symptoms of vaginal discharge and dysuria were included.

Exclusion Criteria

Patients on antifungal treatment and species isolates of *Candida *from the same patient and from the same specimens were not included in the study to avoid duplication of isolates. 

Specimen collection and processing

After obtaining approval from the Institutional Ethics Committee of Krishna Vishwa Vidyapeeth (Deemed to be University) (approval number: KVV/IEC/05/2024), written informed consent was obtained from the participants involved in the research study after the purpose of the study was explained to them. Two HVSs were taken using a speculum inserted into the vagina to separate the vaginal walls. The excess cervical mucus was wiped away with a tissue. A sterile long (22cm) rigid cotton swab was inserted carefully into the posterior fornix, rotated gently, and put into a sterile tube, labelled, and transported to the laboratory [13].

Direct Microscopy

HVSs collected from patients were subjected to microscopy (Gram stain) to screen for the presence of budding yeast cells [14].

Isolation

The swabs received an inoculation on Sabouraud’s dextrose agar (SDA) supplemented with 0.06 mg/ml gentamicin, without cycloheximide (Hi-Media Laboratories, Mumbai, India), and incubated at 37^o^ C for 48-72 hours. The resultant colonies were investigated for growth characteristics suggestive of *Candida *species [13]. Identification of culture growth was by colony characteristics (smooth, white to cream colored colonies were identified as those of *Candida*) [15]. Any growth obtained was correlated with Gram stain findings [3].

Gram Staining

A few colonies from a pure culture were placed onto a clean glass slide to create a smear, which was then subjected to Gram staining. Gram staining revealed round to oval Gram-positive budding cells both with and without pseudohyphae [15].

Identification of Candida Species

The species of *Candida *isolates were identified by standard procedures including germ tube test [16], sugar fermentation test, and growth on CHROM agar [15].

Germ Tube Test

A small number of colonies from pure cultures of *Candida *were treated with 0.5 mL of pooled serum in a test tube and kept at 37°C for two to four hours. After incubation, a drop of suspension was placed on the glass slide, covered with a coverslip, and examined microscopically (Reynolds-Braude phenomenon). The germ tubes were seen as long tube-like projections extending from the yeast cells [17].

Sugar Fermentation Test

Sugar fermentation tests were carried out to differentiate *Candida *species based on their ability to ferment sugars, mainly glucose, maltose, sucrose, lactose, and xylose [13]. Acid production was indicated by a change in the hue of the pH indicator, while the formation of gas was identified by the presence of bubbles in the Durham tube.

CHROMagar Candida Medium

The colony on SDA plates was subcultured onto CHROMagar *Candida *plates and was incubated at 37^ο^C for 24 to 48 hours [18]. HiCrome® *Candida *Differential Agar (HiMedia Laboratories, Mumbai, India) was prepared following the manufacturer’s instructions. *C. albicans*, *C. tropicalis*, *C. krusei*, and *C. glabrata *were identified on CHROMagar *Candida*, based on the characteristic color of the colony.

Statistical analysis

Given the descriptive cross-sectional design of the study, descriptive statistical analysis alone was performed. Microsoft Excel was used to analyze the data and summarize them as frequencies and percentages. Tests of inferential statistics were not conducted.

## Results

A total of 127 *Candida-*positive HVS specimens from women who have clinically diagnosed vaginal candidiasis were analyzed in this study. Of the 127 patients included in the study, the 18-28-year age group accounted for the highest proportion (70; 55.12%), followed by the 29-38-year age group (45; 35.43%) and the 39-45-year age group (12; 9.45%). These findings indicate that vaginal candidiasis was most prevalent among women of reproductive age. The age-wise distribution of the study participants is presented in Table 1.

**Table 1:** Distribution of vaginitis patients with isolated Candida species (n=127) among different age groups n: Number; %: Percentage

Age groups (years)	Total number of patients (n= 127)	Total (%)
18-28	70	55.12
29-38	45	35.43
39-45	12	9.45

**Figure 1:**
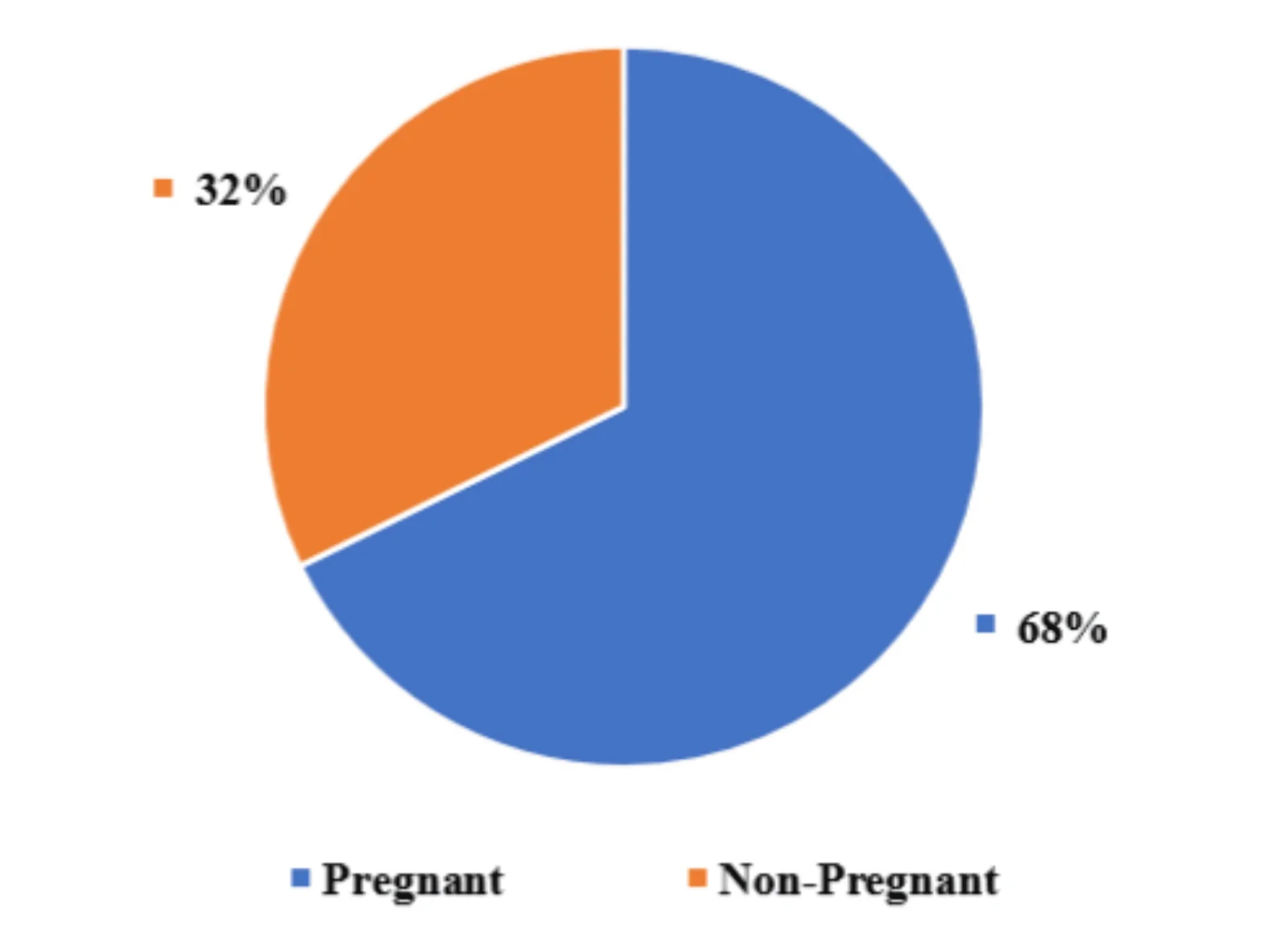
Pregnancy status distribution in vaginitis patients %: Percentage

Among the 127 patients included in the study, 86 (67.72%) were pregnant,and 41 (32.28%) were non-pregnant. The findings indicate that the majority of vaginal candidiasis cases occurred among pregnant women, suggesting a higher burden of infection in this group. The distribution of patients according to pregnancy status is shown in Figure 1.

Microscopic findings

**Figure 2:**
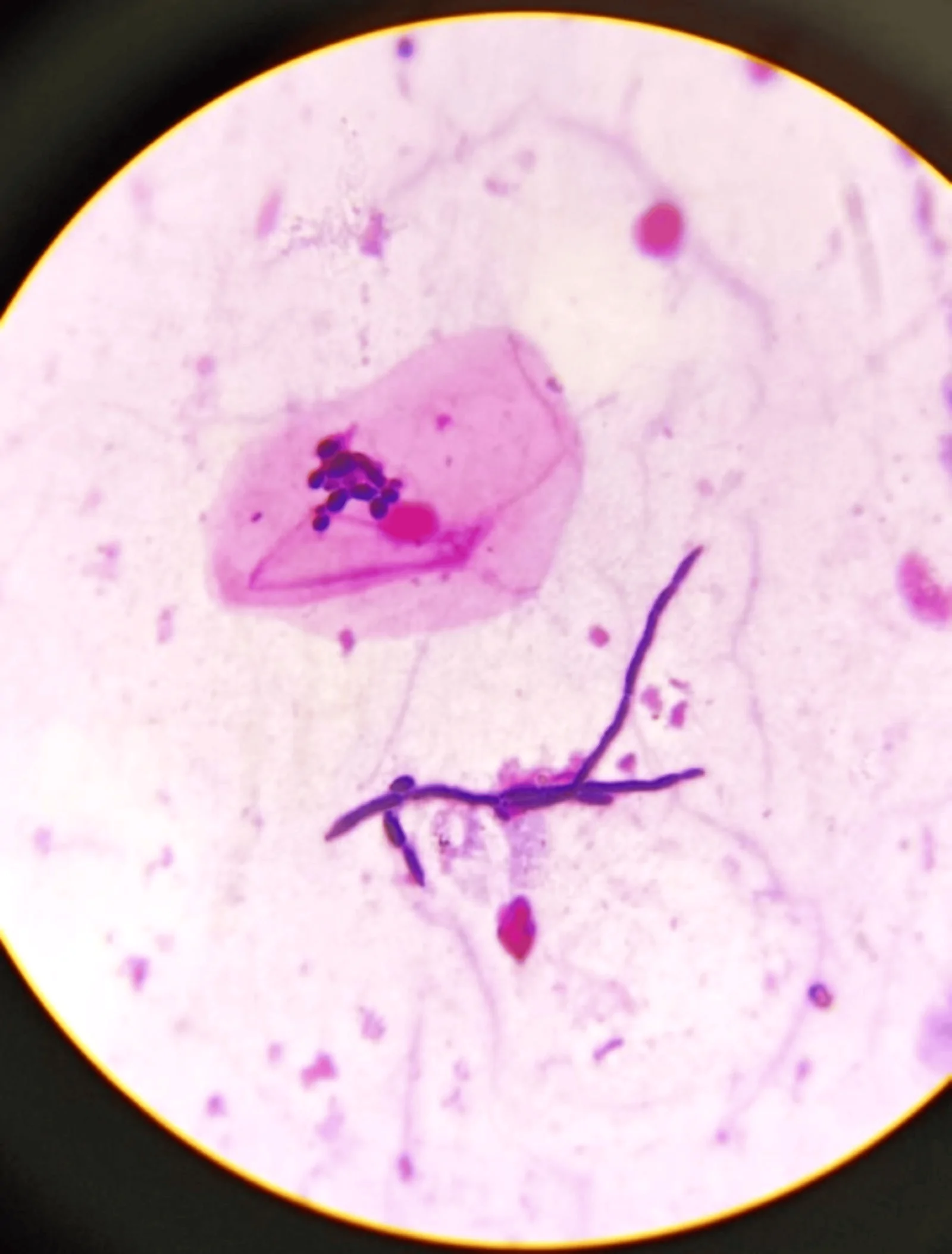
Direct microscopy of high vaginal swab (HVS) (Gram staining × 100)

Direct microscopic examination of the HVS following Gram staining revealed Gram-positive, oval budding yeast cells with pseudohyphae, characteristic of *Candida *species. The presence of budding yeast cells and pseudohyphae provided presumptive evidence of *Candida *infection and supported the clinical diagnosis of vaginal candidiasis. The microscopic appearance is illustrated in Figure 2.

**Figure 3:**
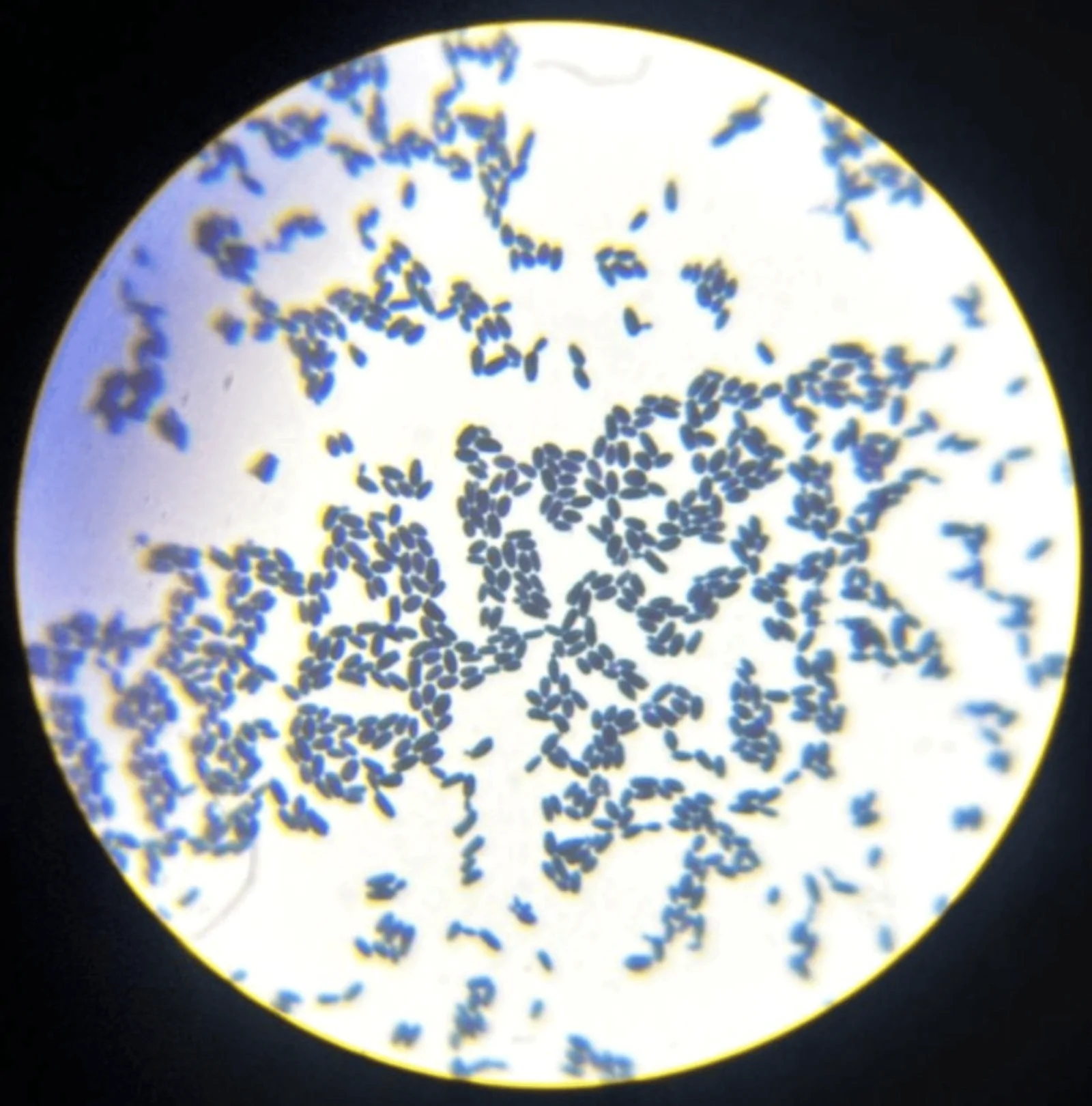
Gram stain of Candida (Gram staining × 100)

Gram staining of the cultured isolates revealed Gram-positive budding yeast cells, consistent with the characteristic morphology of *Candida *species. These microscopic findings supported the preliminary identification of the isolates prior to further phenotypic characterization. A representative Gram-stained preparation is shown in Figure 3.

**Figure 4:**
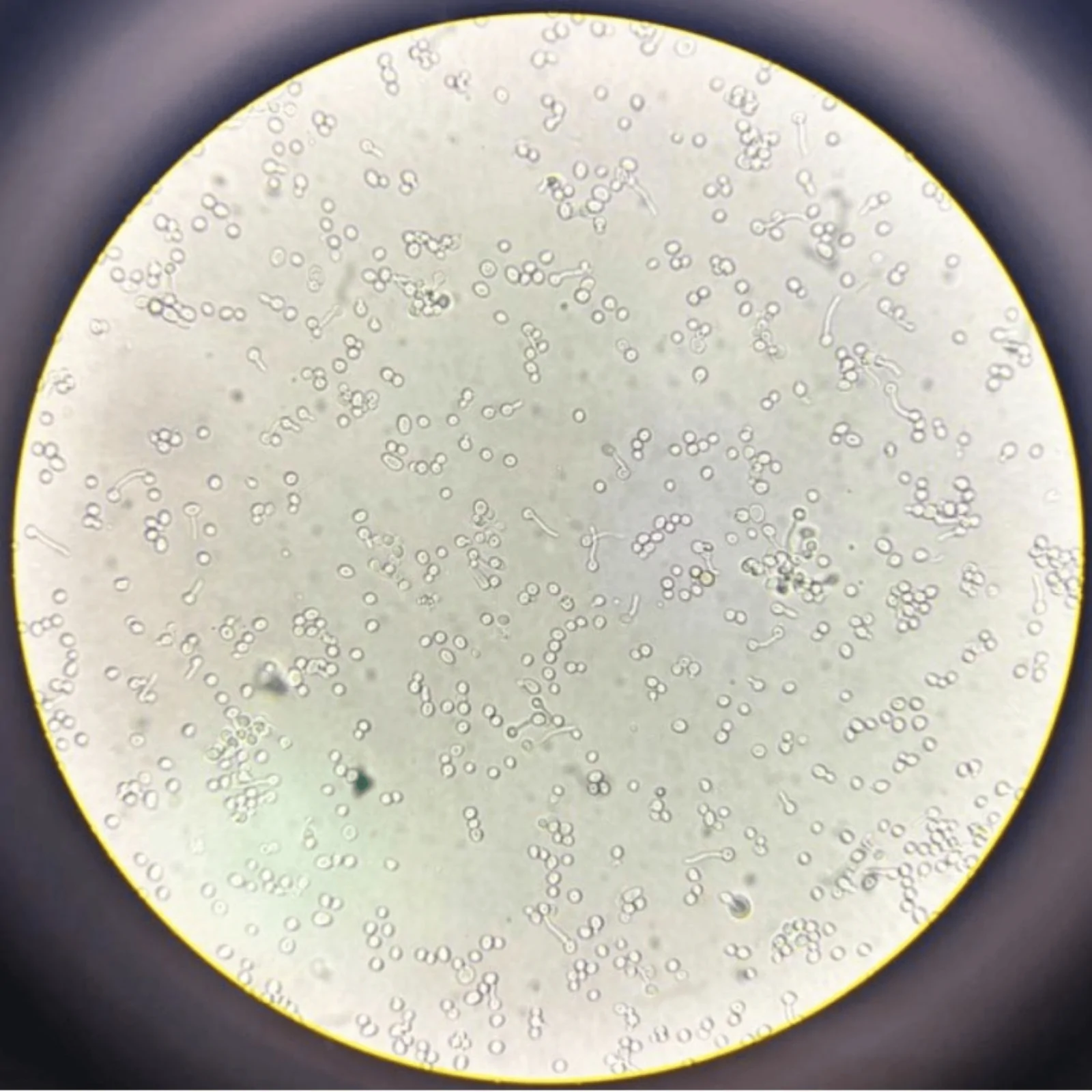
Candida albicans demonstrating germ tube formation (serum ×40)

The isolates were subjected to the germ tube test for the identification of *C. albicans*. Positive isolates demonstrated the formation of germ tubes, characterized by tube-like extensions arising from the yeast cells without constriction at their base, consistent with the typical morphology of *C. albicans*. A representative photomicrograph of the germ tube positive isolate is presented in Figure 4.

Species identification of *Candida *isolates by conventional methods

Carbohydrate fermentation testing further characterized the *Candida *isolates based on their characteristic sugar utilization patterns. All *Candida *species fermented glucose. Sucrose and maltose fermentation were observed in *C. tropicalis*, while *C. albicans* additionally fermented maltose. *C. krusei *and *C. glabrata *exhibited fermentation of glucose only. Because *C. krusei* and *C. glabrata* exhibited identical glucose-only fermentation profiles, differentiation between these species could not be achieved using carbohydrate fermentation testing alone. Lactose and xylose were not fermented by any of the *Candida *species tested. Final species identification was based on the overall identification algorithm described in the Methods section, including the characteristic colony morphology on CHROMagar *Candida *and the other identification procedures, while carbohydrate fermentation testing was used as a complementary identification method. The final *Candida *species distribution was 60 (47.24%) *C. albicans*, 32 (25.20%) *C. krusei*, 21 (16.54%) *C. tropicalis*, and 14 (11.02%) *C. glabrata*, as shown in Table 2.

**Table 2:** Species distribution and carbohydrate fermentation patterns of Candida isolates The table shows the frequency and percentage distribution of the identified Candida species along with their respective carbohydrate fermentation profiles. Variations in carbohydrate fermentation reactions were observed among the different species. G: glucose; S: sucrose; L: lactose; M: maltose; X: xylose; AG: acid and gas production; -: no fermentation (negative reaction); n: Number; %: percentage.

G	S	M	L	X	Identified Candida species (n)	Total %
AG	-	AG	-	-	C. albicans (60)	47.24
AG	-	-	-	-	C. krusei (32)	25.20
AG	AG	AG	-	-	C. tropicalis (21)	16.54
AG	-	-	-	-	C. glabrata (14)	11.02

**Figure 5:**
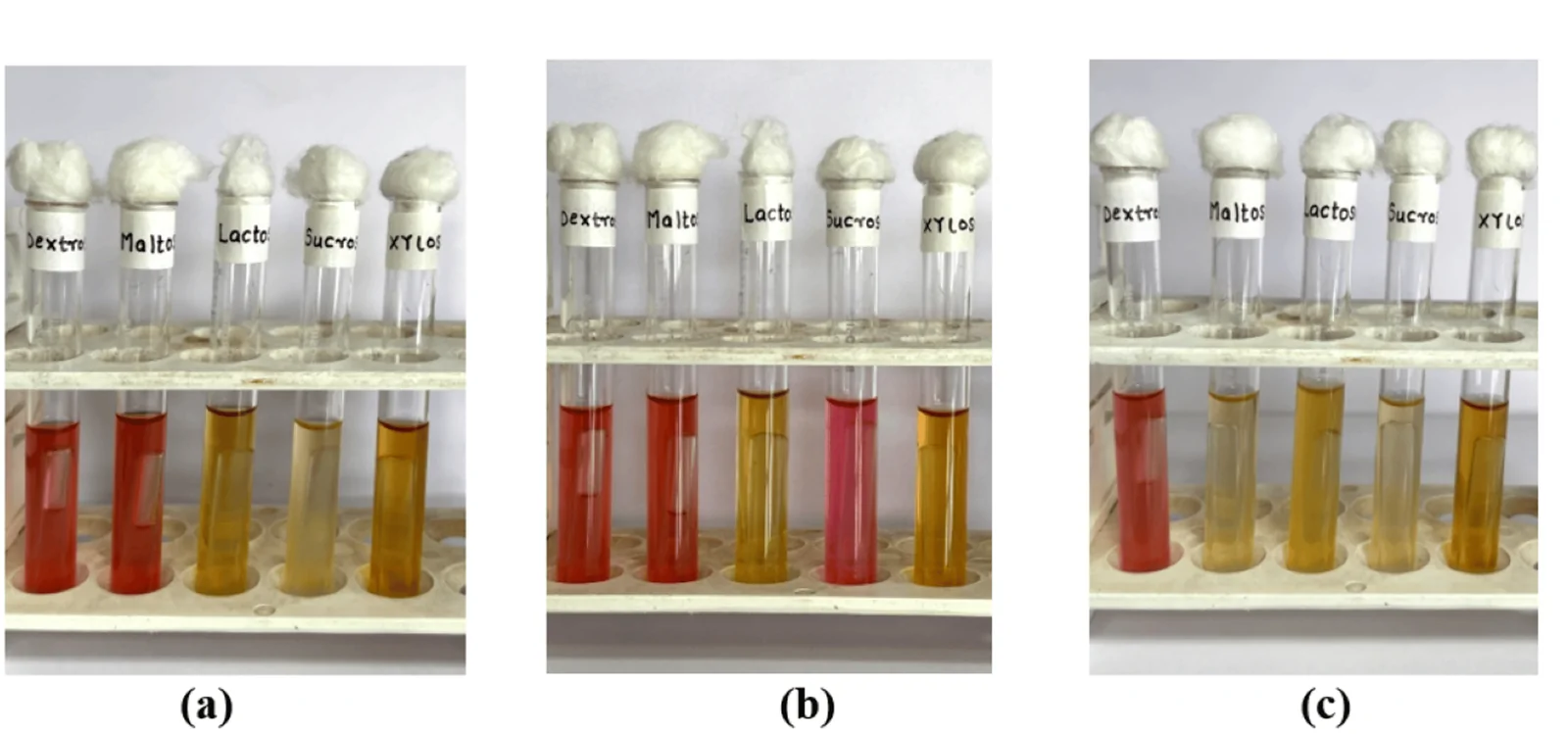
Carbohydrate fermentation profiles of different Candida species (a) *Candida albicans*, (b) *Candida tropicalis*, and (c) *Candida krusei* and *Candida glabrata*

The identified *Candida *isolates were subjected to carbohydrate fermentation testing to characterize their biochemical profiles. The fermentation results were interpreted as supportive findings alongside CHROMagar *Candida*, which was used for presumptive species identification based on colony color and morphology. The representative carbohydrate fermentation patterns are presented in Figure 5.

Chromogenic differentiation of *Candida *species using CHROMagar *Candida*

**Figure 6:**
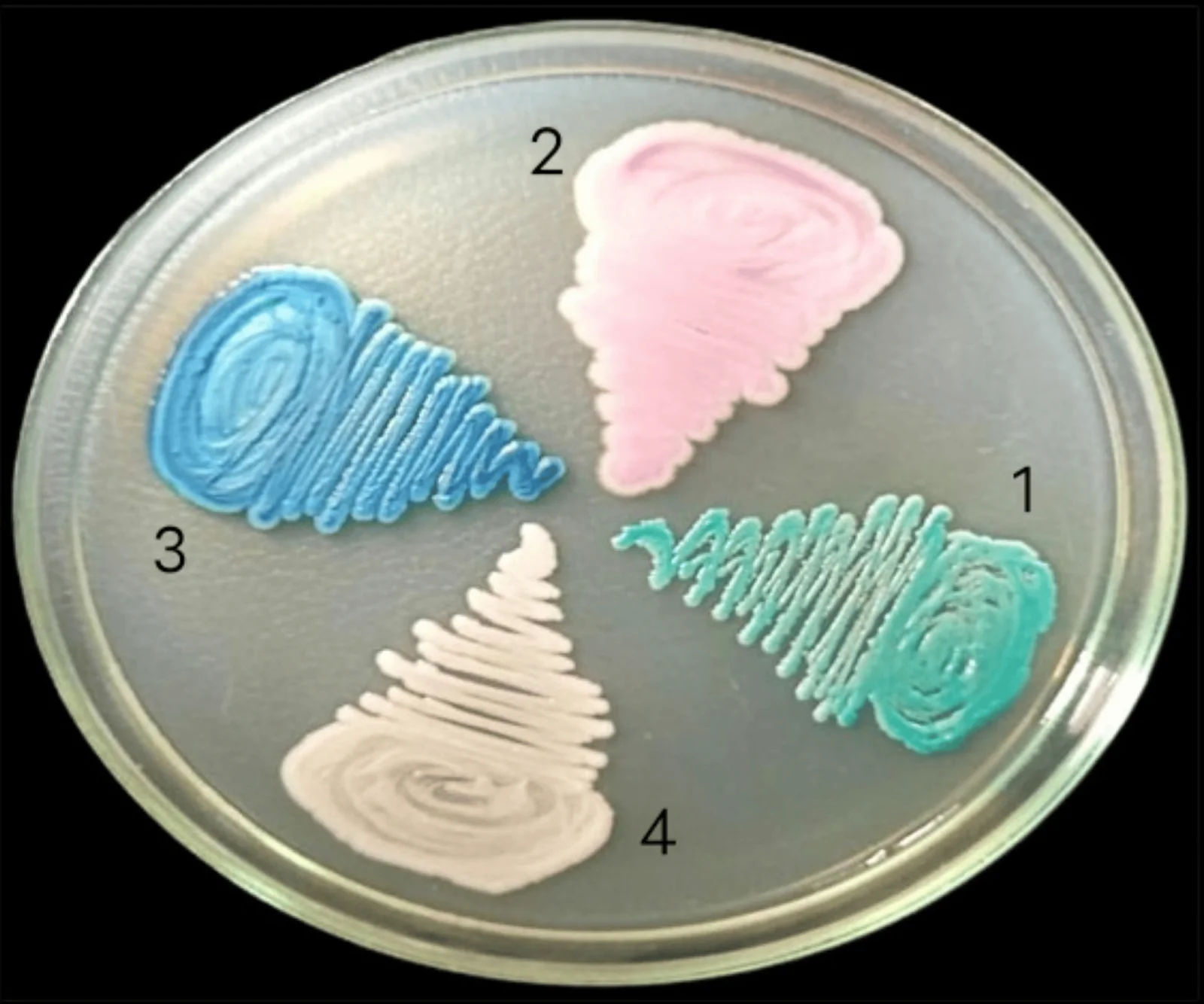
CHROMagar Candida illustrating characteristic species-specific colony coloration of Candida isolates 1. *Candida albicans* appearing as light green colonies; 2. *Candida krusei* appearing as purple fuzzy colonies; 3. *Candida tropicalis* appearing as metallic blue colonies; 4. *Candida glabrata* appearing as white to cream colonies.

HiCrome® *Candida *Differential Agar (HiMedia Laboratories, Mumbai, India) demonstrated characteristic colony morphology and pigmentation among the *Candida *isolates. Light green, smooth colonies were observed for C. albicans, blue to metallic blue colonies for C. tropicalis, purple, fuzzy colonies for C. krusei, and cream to white, smooth colonies for C. glabrata, as shown in Figure 6. The colony morphology and pigmentation were interpreted according to the manufacturer's guidelines and were used for presumptive differentiation of *Candida *species.

Table 3 summarizes the identified *Candida *species classified according to their characteristic colony coloration on CHROMagar *Candida *medium. The distinct color and morphological characteristics of the colonies enabled the presumptive differentiation of the isolates. Representative colony appearances of the various *Candida *species on CHROMagar *Candida *medium are shown in Figure 6.

**Table 3:** The variety of Candida species based on characteristic colony coloration on CHROMagar Candida medium The variety of Candida species on CHROMagar Candida medium is summarized in Table 3. Species differentiation was achieved based on characteristic colony coloration, containing (1) *C. albicans* appearing as light green colonies, (2) *C. krusei *as purple fuzzy colonies, (3) *C. tropicalis *as metallic blue colonies, and (4) *C. glabrata *as white to cream colonies. The frequency of distinct isolates observed for each individual species is reported. n: Number

Color of colonies (pigmentation) on CHROMagar Candida	Number of Candida isolates identified (n)
Light green	C. albicans ^1^(60)
Purple (fuzzy)	C. krusei ^2^ (32)
Metallic blue	C. tropicalis ^3^ (21)
White to cream	C. glabrata ^4^ (14)

**Figure 7:**
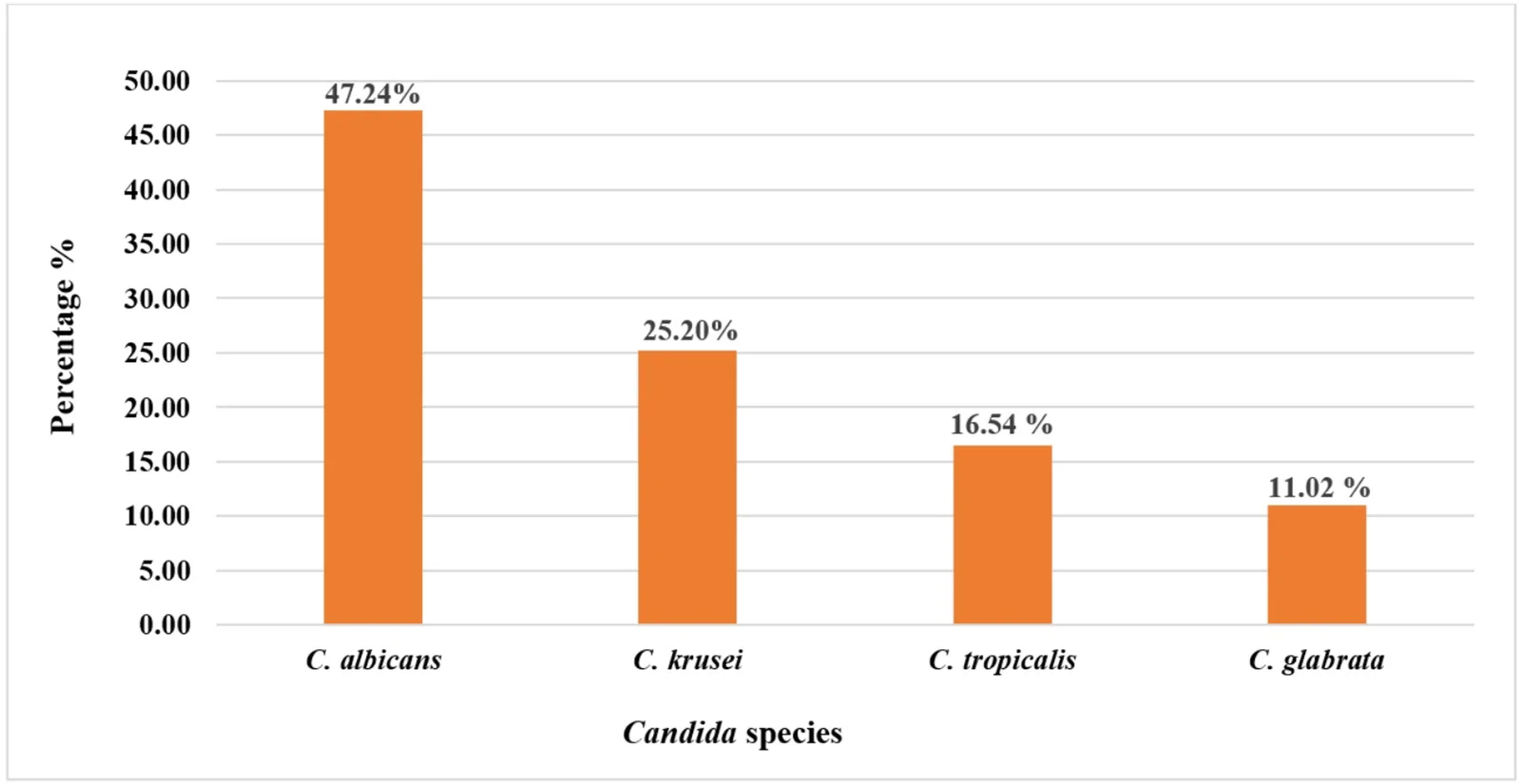
Species distribution of Candida isolated from specimens %: Percentage

Among the 127 culture-positive *Candida *isolates, *C. albicans* was the most frequently isolated species, accounting for 60 (47.24%) isolates. This was followed by *C. krusei*, which was isolated from 32 (25.20%) samples. *C. tropicalis *were identified in 21 (16.54%) samples and *C. glabrata* were identified in 14 (11.02%) samples, respectively. Overall, *C. albicans *remained the predominant species isolated, whereas NAC* *species collectively constituted a substantial proportion of the isolates. The species-wise distribution of *Candida *isolates is illustrated in Figure 7.

Carbohydrate fermentation testing and CHROMagar *Candida *(HiMedia Laboratories, Mumbai, India) medium were used as complementary phenotypic methods for the identification and characterization of clinical *Candida *isolates. Carbohydrate fermentation testing provided supportive biochemical profiles, while CHROMagar *Candida *(HiMedia Laboratories, Mumbai, India) provided presumptive species differentiation based on characteristic colony color and morphology.

## Discussion

The present study assessed the epidemiological profile and species distribution of *Candida *isolates recovered from women presenting with vaginitis at a tertiary care center. The findings demonstrate that VVC remains a significant cause of vaginitis, with a predominance among young reproductive-age women and a notable burden of NAC* *species. VVC continues to be among the most common causes of vaginal infection among reproductive-age women. In this study, *Candida *species were isolated from 127 (29.67%) of 428 women presenting with symptoms of vaginitis. This finding is comparable to previous reports indicating that *Candida *is responsible for a significant percentage of vaginal infections among symptomatic women. Tafazoli et al. [19] stated that culture confirmed *Candida *infection in 68 out of 163 women evaluated for vaginal candidiasis, emphasizing the continued burden of *Candida *infections in gynecological practice. The slight variation in prevalence between studies may be attributed to differences in study population, geographical location, diagnostic methods, and associated risk factors among the participants.

The majority of *Candida-*positive cases in the current study occurred among women aged 18-28 years (70; 55.12%). This result aligns with the outcomes of Irene et al., who reported the highest prevalence of VVC among women who are of reproductive age (18-35 years) [20]. The increased prevalence in younger women may be attributed to hormonal activity, increased glycogen deposition in vaginal epithelial cells, sexual activity, and other host-related factors that promote *Candida *colonization. Variations in age-specific prevalence reported across studies may also reflect differences in healthcare-seeking behavior and demographic characteristics of the study populations.

In our study, 86 (67.72%) of the 127 *Candida-positive* women were pregnant. This finding is comparable in context with the observations of Ali et al., who reported a 61.4% prevalence of *Candida *species among pregnant women[21], although direct comparison is limited by differences in denominators and study populations. Similar observations were reported by Khan et al. [22], who described pregnancy as a vulnerability to VVC and observed a high incidence of *Candida *infection among preterm mothers who attend antenatal clinics. Purnamasari et al. discovered a high frequency of NAC* *species that induce VVC during pregnancy [23]. The rising number of pregnancy-related VVC cases caused by NAC has made diagnosing and treating VVC more challenging. Increased amounts of estrogen during pregnancy increase glycogen concentration within vaginal tissues, creating a favorable environment for *Candida *growth and colonization. Differences in the prevalence of pregnancy-associated VVC among studies may be influenced by variations in antenatal care practices, socioeconomic factors, and regional epidemiological characteristics.

Regarding species distribution, *Candida albicans *was the most frequently isolated species, accounting for 60 (47.24%) of all isolates. This finding agrees with the research conducted by Khan et al., who stated that *Candida albicans* accounted for 41.7% of isolates [22], and Tafazoli et al., who documented a prevalence of 59.7% [19]. Although *C. albicans* remains the predominant pathogen, its proportion in our investigation was less than that reported in many earlier studies, suggesting a gradual epidemiological shift. This variation may reflect differences in geographic distribution, patient characteristics, prior antifungal exposure, and local epidemiological trends.

A noteworthy result of the current investigation was the predominance of NAC species, which accounted for 67 (52.76%) of all isolates. This observation is consistent with the growing body of evidence indicating a shift in the epidemiology of VVC from *Candida albicans *toward NAC species. Similar results have been documented by George Aboagye et al. [9], Tafazoli et al. [19], and Badiee et al. [24], who highlighted the increasing isolation of species such as *C. krusei*, *C. tropicalis*, and *C. glabrata* and their clinical relevance due to being less susceptible to widely used antifungal agents. The increasing recovery of NAC* *species observed across different regions suggests an evolving epidemiological pattern that warrants continued surveillance and accurate species-level identification.

Among the NAC isolates identified, *C. krusei *was the most prevalent species in the current study, accounting for 32 (25.20%) isolates, followed by *C. tropicalis *(21; 16.54%) and *C. glabrata* (14; 11.02%). Similar patterns have been documented by Tafazoli et al. [19], Khan et al. [22], and Bitew et al. [25]. The relatively higher proportion of *C. krusei *observed in the present study may reflect differences in the study population and local or regional epidemiological patterns. In the present study, species identification was based on complementary phenotypic methods, with carbohydrate fermentation providing supportive biochemical profiles and CHROMagar *Candida *providing presumptive differentiation based on colony color and morphology. As *C. krusei *and *C. glabrata* showed similar glucose-only fermentation profiles, their differentiation was based on the complementary phenotypic characteristics observed on CHROMagar *Candida*. The predominance of *C. krusei* remains clinically relevant because of its intrinsic resistance to fluconazole and highlights the importance of accurate species identification for appropriate clinical management.

The prevalence of NAC species has important therapeutic implications, as these organisms frequently demonstrate decreased susceptibility to azole antifungals. Previous studies, including those by Khan et al. [22] and Badiee et al. [24], have documented that *Candida *isolates are increasingly resistant to antifungals and emphasized the demand for routine species-level identification and periodic surveillance of antifungal susceptibility patterns. These protocols are crucial for guiding appropriate therapy and minimizing treatment failure and recurrence.

In the present study, species-level identification of *Candida *isolates was analyzed using established phenotypic methods; this includes the glucose fermentation test and the germ tube test. Despite their widespread use in diagnostic laboratories, these approaches are relatively time-consuming and may have limited specificity when species exhibit similar fermentation patterns. Conversely, CHROMagar *Candida *facilitated rapid presumptive identification based on characteristic colony pigmentation. The identification results obtained using CHROMagar *Candida *were largely comparable to those obtained by conventional phenotypic methods, indicating that CHROMagar *Candida *is a valuable adjunct for the timely and reliable identification of *Candida *species. These findings support the utility of CHROMagar *Candida *as a practical supplementary method for routine laboratory identification of clinically important *Candida *species.

The predominance of NAC species observed in the present study highlights the changing epidemiology of VVC. As several NAC* *species differ in their pathogenic potential and therapeutic response, accurate species-level identification remains essential for appropriate laboratory diagnosis and effective clinical management. The findings of the present study emphasize the importance of continuous epidemiological surveillance to monitor changing species distribution and support evidence-based management of VVC.

Limitations

Certain limitations of the present study should be considered when interpreting the findings. Antifungal susceptibility testing was beyond the scope of the present study; therefore, resistance patterns of the identified *Candida *species were not evaluated. In addition, molecular methods for species confirmation were not employed, and species identification relied on conventional phenotypic methods and CHROMagar *Candida *medium. Although these methods are widely used in routine microbiology laboratories, the absence of molecular confirmation may have affected the accuracy of species identification, particularly for closely related NAC* species such as C. krusei *and *C. glabrata*. Furthermore, the study was conducted at a single tertiary care center with a relatively limited sample size, which may restrict the generalizability of the findings to other populations. Clinical risk factors associated with VVC, patient treatment outcomes, and recurrence rates were also not evaluated, limiting the assessment of the broader clinical significance of the identified *Candida *species. Future multicenter studies involving larger study populations, molecular identification techniques, and antifungal susceptibility testing are warranted to provide a more comprehensive understanding of the epidemiology and clinical management of VVC.

Despite these limitations, the present study provides important insights into the epidemiology and species distribution of *Candida *among women with vaginitis. The combined use of conventional phenotypic methods and CHROMagar *Candida *enabled a comparative evaluation of species identification techniques and demonstrated the practical utility of chromogenic media for rapid presumptive identification of clinically important *Candida *species. These findings contribute to the existing literature and provide baseline epidemiological data that may support future surveillance studies and improvements in routine laboratory diagnosis.

## Conclusions

The present study demonstrated that VVC was most common among women of reproductive age, particularly during pregnancy. Although *Candida albicans* remained the most common individual species isolated, NAC species collectively accounted for a higher proportion of isolates, highlighting the substantial burden of NAC species in the study population. The substantial prevalence of *C. krusei*, *C. tropicalis*, and *C. glabrata *emphasizes the necessity of routine species-level identification and continuous surveillance of trends in antifungal sensitivity to ensure appropriate therapy and improve patient outcomes. The recognition of species of *Candida *by conventional microbiological methods can be labor-intensive and time-consuming, often necessitating the use of several complementary tests. The incorporation of CHROMagar *Candida *into routine laboratory workflows allows for rapid presumptive species differentiation based on distinct chromogenic reactions, enhancing diagnostic accuracy and reducing the time required for preliminary identification.

Precise identification and description of *Candida *species play a major part in the diagnosis of vaginitis and may help with improved clinical care. The present investigation offers important details about the range of *Candida *species that were separated from vaginal samples. Future research using bigger sample sizes and testing for antifungal susceptibility is recommended to further expand the understanding of *Candida *epidemiology and guide evidence-based therapeutic approaches.

## References

[1] Hösükoğlu FG, Ekşi F, Erinmez M, Uğur MG (2022). An epidemiologic analysis of vulvovaginal candidiasis and antifungal susceptibilities. Infect Microb Dis.

[2] Weldegebreal F, Negesa AS, Ayana DA (2025). Bacterial vaginosis, vaginal Candida colonization and antifungal susceptibility patterns in pregnant women of eastern Ethiopia: a prospective study. Sci Rep.

[3] Devhare D (2021). Species distribution and antifungal susceptibility pattern Candida species isolated from patients with vulvovaginal candidiasis by Vitek 2 compact system, in Western Maharashtra. IOSR J Dent Med Sci.

[4] Yesudhason BL, Mohanram K (2015). Candida tropicalis as a predominant isolate from clinical specimens and its antifungal susceptibility pattern in a tertiary care hospital in Southern India. J Clin Diagn Res.

[5] Mullick JB, Majumdar T, Ray J, Sil SK (2015). Changing trends of Candida isolates and their antifungal susceptibility pattern in vulvovaginal candidiasis cases of Tripura, North East India. J Evol Med Dent Sci.

[6] Tulasidas S, Rao P, Bhat S, Manipura R (2018). A study on biofilm production and antifungal drug resistance among Candida species from vulvovaginal and bloodstream infections. Infect Drug Resist.

[7] Shrestha P, Pokharel SM, Shrestha A (2020). Antifungal susceptibility pattern of Candida isolates causing vulvovaginitis in reproductive age women. Tribhuvan Univ J Microbiol.

[8] Ganeshkumar A, Nagarajan P, Mahalingam P, Balasubramanian S, Archunan PA, Govindaraju A, Rajaram R (2020). Antifungal susceptibility and virulence profile of candida isolates from abnormal vaginal discharge of women from southern India. Eur J Obstet Gynecol Reprod Biol.

[9] Aboagye G, Waikhom S, Asiamah EA (2025). Antifungal susceptibility profiles of Candida and non-albicans species isolated from pregnant women: implications for emerging antimicrobial resistance in maternal health. Microbiol Spectr.

[10] Mahmoudi Rad M, Zafarghandi S, Abbasabadi B, Tavallaee M (2011). The epidemiology of Candida species associated with vulvovaginal candidiasis in an Iranian patient population. Eur J Obstet Gynecol Reprod Biol.

[11] Lillis RA, Parker RL, Ackerman R (2023). Clinical evaluation of a new molecular test for the detection of organisms causing vaginitis and vaginosis. J Clin Microbiol.

[12] Rudresh SM, Nikhil V, Shakuntala PN (2024). Antifungal susceptibility profile of Candida species isolated from women with vulvovaginal candidiasis. J Lab Physicians.

[13] Babin D, Kotigadde S, Rao PS, Rao TV (2013). Clinico-mycological profile of vaginal candidiasis in a tertiary care hospital in Kerala. Int J Res Biol Sci.

[14] Kombade SP, Abhishek KS, Mittal P, Sharma C, Singh P, Nag VL (2021). Antifungal profile of vulvovaginal candidiasis in sexually active females from a tertiary care hospital of Western Rajasthan. J Family Med Prim Care.

[15] Siddiqui R (2019). Clinical patterns and risk factors of vulvo-vaginal candidiasis among women of reproductive age attending a tertiary hospital in central India. Stamford J Microbiol.

[16] Mohanty S, Xess I, Hasan F, Kapil A, Mittal S, Tolosa JE (2007). Prevalence & susceptibility to fluconazole of Candida species causing vulvovaginitis. Indian J Med Res.

[17] Chander J (2018). Textbook of Medical Mycology. 4th ed.. Textbook of Medical Mycology. 4th ed..

[18] Baradkar VP, Mathur M, Kumar S (2010). Hichrom candida agar for identification of Candida species. Indian J Pathol Microbiol.

[19] Tafazoli M, Gholami M, Mohebbi-Dehnavi Z, Shaghaghi F, Kamali Z (2021). Determining the frequency of Candida species in women with candidal vaginal infection frequency of Candida species in women with candida vaginal infection. J Educ Health Promot.

[20] Irene VR, Sajeeth CI, Karthikeyan V, Sabitha J (2023). Assessment of risk factors for developing Vulvovaginal Candidiasis among women at various age groups. Biosci Biotechnol Res Asia.

[21] Ali M, Edrees WH, Al-Shehari WA (2024). Antifungal susceptibility pattern of Candida species isolated from pregnant women. Front Cell Infect Microbiol.

[22] Khan M, Ahmed J, Gul A, Ikram A, Lalani FK (2018). Antifungal susceptibility testing of vulvovaginal Candida species among women attending antenatal clinic in tertiary care hospitals of Peshawar. Infect Drug Resist.

[23] Purnamasari I, Ervianti E, Damayanti D (2022). Candida species isolated from patient with vulvovaginal candidiasis in pregnancy. Berk Ilmu Kesehat Kulit Kelamin.

[24] Badiee P, Boekhout T, Haddadi P (2022). Epidemiology and antifungal susceptibility of Candida species isolated from 10 tertiary care hospitals in Iran. Microbiol Spectr.

[25] Bitew A, Abebaw Y (2018). Vulvovaginal candidiasis: species distribution of Candida and their antifungal susceptibility pattern. BMC Womens Health.

